# Describing Treatment Patterns for Elderly Patients with Intrahepatic Cholangiocarcinoma and Predicting Prognosis by a Validated Model: A Population-Based Study

**DOI:** 10.7150/jca.53978

**Published:** 2021-03-30

**Authors:** Hanlong Zhu, Kun Ji, Wei Wu, Si Zhao, Jian Zhou, Chunmei Zhang, Ruiyi Tang, Lin Miao

**Affiliations:** 1 Medical Centre for Digestive Diseases, the Second Affiliated Hospital of Nanjing Medical University, Nanjing 210011, Jiangsu Province, China.; 2 Department of Interventional Radiology, the First Affiliated Hospital of Zhengzhou University, Zhengzhou 450052, Henan Province, China.; 3 Department of Medical Oncology, the First Affiliated Hospital, School of Medicine, Zhejiang University, Hangzhou 310003, Zhejiang Province, China.

**Keywords:** geriatric, intrahepatic cholangiocarcinoma, treatment patterns, nomogram, SEER.

## Abstract

**Background:** Elderly patients with Intrahepatic Cholangiocarcinoma (ICC) are frequently under-represented in clinical trials, which leads to the unclear management of ICC in elderly patients. This study aimed to describe treatment patterns and establish a reliable nomogram in elderly ICC patients.

**Methods:** Based on the Surveillance, Epidemiology, and End Results (SEER) database, we conducted a retrospective analysis of 1651 elderly patients (≥65 years) diagnosed with ICC between 2004 and 2016.

**Results:** For the whole study population, 29.3% received only chemotherapy, 26.7% no tumor-directed therapy, 19.1% surgery alone, 17.5% radiotherapy, and 7.4% surgery plus chemotherapy. Compared with the age group of 65-74 years, patients aged ≥75 years were less likely to accept treatment. Among patients 66-74 years of age, surgery alone resulted in a median overall survival (OS) of 30 months, surgery combined with chemotherapy 26 months, radiotherapy 17 months, chemotherapy alone 10 months and no therapy 3 months; while among patients ≥75 years of age, the median OS was 21, 25, 14, 9 and 4, respectively. Moreover, independent prognostic indicators including age, gender, grade, tumor size, T stage, N stage, M stage, and treatment were incorporated to construct a nomogram. The C-indexes of the OS nomogram were 0.725 and 0.724 for the training and validation cohorts, respectively. Importantly, the predictive model harbored a better discriminative power than the American Joint Committee on Cancer TNM staging system.

**Conclusion:** Active treatment should not be abandoned among all the elderly patients with ICC. The validated nomogram provided an effective and practical tool to accurately evaluate prognosis and to guide personalized treatment for elderly ICC patients.

## Introduction

Cholangiocarcinoma (CCA) represents a group of heterogeneous neoplasms derived from the epithelium within the biliary tree, accounting for approximately 3% of all gastrointestinal malignancies 1. According to the site of anatomic origin, CCA is generally classified as intrahepatic, perihilar, or distal 1, 2. Intrahepatic cholangiocarcinoma (ICC), the second most common form of primary liver cancers, is localized to the area between the small intrahepatic ductules and the second-order bile ducts 2-4. The incidence of ICC has been on the rise over the past few decades, especially in western countries 5; for instance, 5000-8000 new cases were reported annually in the United States^6^. Unfortunately, despite the rarity of this tumor, it inclines to be terminal or even fatal when diagnosed 3. Furthermore, most reports demonstrated that the overall survival of ICC has not improved in recent years, with a 5-year survival rate of less than 5% 5. It is worth noting that the prevalence of ICC grows with age and the rate of elderly patients is almost twice that of young patients 7. Although ICC has recently been found in increasingly younger patients, the most common age range for ICC is 55 to 75 years old^3^, further indicating a large proportion of ICC patients are elderly. However, the older patients were often under-represented in clinical trials of cancer therapy, which results in relatively limited information instructing the clinical decision-making 8. In view of this, it is particularly crucial for the elderly ICC to study the treatment pattern and its impact on the prognosis of patients in actual clinical practice.

Currently, hepatectomy with histologically negative margins remains the only well-established treatment option for localized or resectable ICC which offers the best possibility of cure 3, 9. As for patients with advanced or unresectable disease, the use of combined chemotherapy (gemcitabine and cisplatin) is recommended as the standard therapy by the ABC-02 study 10. Nevertheless, these data should be cautiously extrapolated to the elderly patients owing to their reduced performance status, worse nutritional status, decreased functional reserve, as well as more preexisting comorbidities 7. Several previous research studies have revealed that elderly patients undergoing hepatic surgery had a higher rate of postoperative complications and mortality than the younger counterparts 11, 12. Consequently, treatment decisions depended heavily on the age of the patient as elderly populations were relatively conservative in choosing aggressive treatments compared with younger patients, which was in accordance with prior observation 13. On the contrary, it was reported that age alone was not a contraindication for hepatic resection 7, and the long-term survival was found to be comparable between the elderly and younger patients receiving surgical resection 7, 14. Moreover, elderly patients were less tolerant to chemotherapy and more susceptible to toxic reactions 15, which might place them at an increased risk of chemotherapy. Thus, chemotherapy was less likely to be chosen for older patients with poor physical condition 16. Yet a recent study indicated that age alone was insufficient to influence decisions on chemotherapy 17. Given these controversies, the optimal management of elderly ICC patients is still not understood, which requires a good balance between the patient prognosis and their capability to withstand cancer treatment.

The accuracy of prognostic estimation is essential to guide individualized therapeutic strategies. So far, the American Joint Committee on Cancer (AJCC) TNM staging system has been widely applied to assess the outcomes of ICC patients. However, other clinical/pathological factors such as age 18, gender 17, tumor differentiation 7, 19, tumor diameter 19, and therapy method 9 can also affect the prognosis of patients. Therefore, the prediction model combining the prognostic clinicopathological parameters may not only evaluate the outcome more accurately, but also facilitate clinical decision-making. As an integrative and visualized predictive model, nomogram has been developed in a variety of cancers 20-22. But, to our knowledge, a nomogram that predicts the survival in elderly patients with ICC has not yet been constructed. Besides, the prospective clinical trial on this topic is lacking. Accordingly, we undertook this study based on the Surveillance, Epidemiology, and End Results (SEER) database to fill this knowledge gap by providing more representative and informative evidence. This population-based research aimed to delineate treatment patterns and outcomes in elderly ICC patients, and to establish a nomogram for individual survival prediction.

## Material and Methods

### Data Source and Study Population

**Figure 1:**
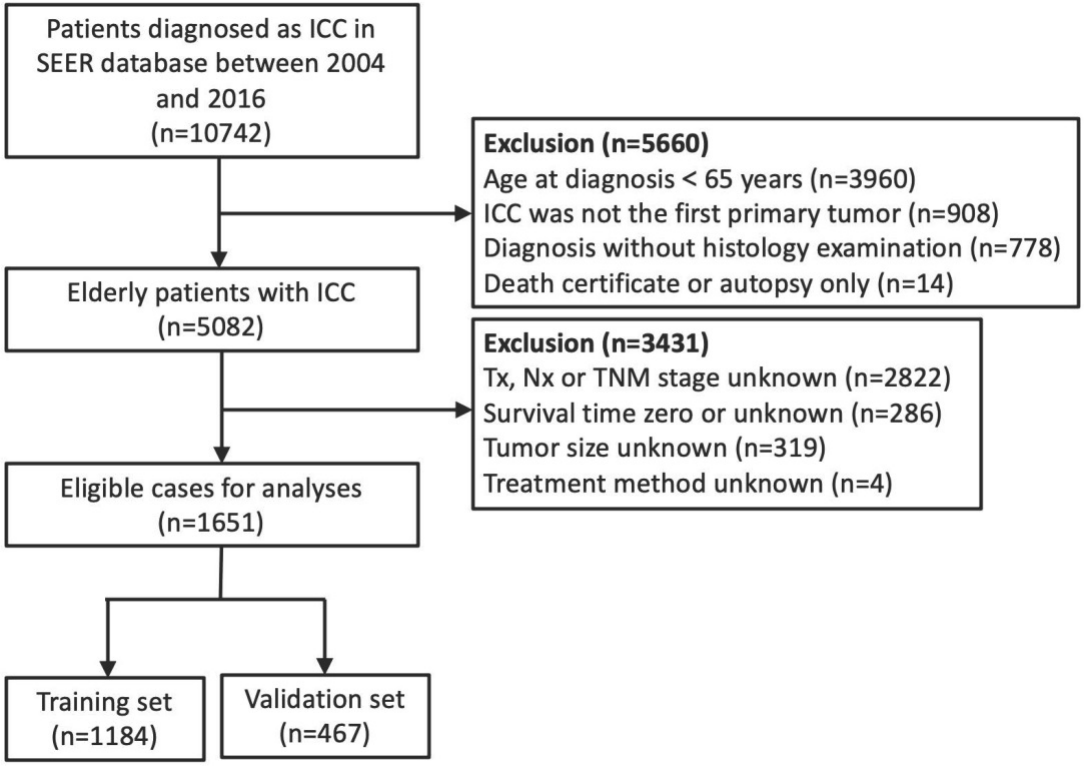
Flow diagram of eligible elderly patients diagnosed with intrahepatic cholangiocarcinoma.

The approval of the institutional review board was not necessary for this study because data from the SEER database are publicly available. The SEER program, funded by the National Cancer Institute (NCI), collects information on cancer patients in 19 geographic regions of the U.S., accounting for about 34% of the U.S. population 23. We used the SEER database to identify elderly patients diagnosed with ICC between 2004 and 2016. The SEER*Stat software (version 8.3.6; NCI, Bethesda, MD) was utilized to obtain per-patient data. Only cases that met the following preassigned criteria were eligible for inclusion: (1) age 65 or older at diagnosis; (2) microscopically/histologically-confirmed ICC as its first and only malignant tumor; (3) histology code 8140 (adenocarcinoma) combined with primary site code C22.1 (intrahepatic bile duct), or histology codes 8160 (cholangiocarcinoma) combined with primary site code C22.0 (liver) or C22.1 based on the International Classification of Diseases for Oncology, 3rd Edition (ICDO-3); (4) known treatment modalities and survival data description; (5) complete information on age, race, gender, tumor size, TNM staging; and (6) ICC diagnosis not determined by autopsy or death certificate. We excluded patients with a follow-up period of less than 1 month owing to the limited immortal time bias 24. Ultimately, a total of 1651 elderly ICC patients were selected in the analysis, 70% of whom were randomly assigned into the training set (n= 1184) for nomogram construction, while the rest constituted the validation set (n= 467) for internal verification. Figure 1 summarizes the detailed process of patient selection.

### Study Variables

The information for each cohort member was retrospectively collected, including demographic characteristics (age, race, gender, and marital status), tumor-related parameters [tumor size, histological grade, and American Joint Committee on Cancer (AJCC) TNM stage], type of first-line treatment received, and follow-up data (vital status, and survival time). The continuous variables were classified to match the nomogram. According to the optimal cut-off value, participants in this research were divided into two groups: 65‐74 years old and ≥75 years old. In order to maximize predictive power, the tumor size was grouped into four categories: ≤2.0 cm, 2.1-5.0 cm, 5.1-10.0 cm, and >10.0 cm. Similarly, the categorical variables were stratified in the light of clinical reality. The treatment was categorized into five sets: (1) surgery alone; (2) chemotherapy alone; (3) any radiotherapy (with or without other forms of treatment); (4) surgery and chemotherapy; and (5) no tumor-directed therapy. Overall survival (OS) was the principal outcome of this study, which was calculated from the date of confirmed diagnosis until the date of death or last follow-up.

### Development and Validation of Nomogram Model

Both univariate and multivariate Cox proportional hazards regression analyses were conducted to screen out covariates (P < 0.05) that significantly affected OS in the training cohort. Based on these identified independent prognostic factors, the model of nomogram was established to predict 3- and 5-year OS in elderly ICC patients. The performance of the prediction model was assessed by both discrimination and calibration measurements. Specifically, the concordance index (C-index) was adopted to evaluate the discriminative ability of the predictive model, which quantifies the discrepancies between observed and predicted outcomes 25. And higher C-index value means a more accurate prediction. Additionally, calibration plots were applied to reflect the consistency between the actual survival and predicted probabilities, and a calibration curve along the 45‐degree diagonal line indicates that the model is perfect 26. The survival prediction between the traditional AJCC staging system and the nomogram was undertaken via the area under receiver operating characteristic (ROC) curve (AUC). AUC was computed severally from the first month to the fifth year (sixtieth month). We also carried out decision curve analysis (DCA) to determine the clinical value and benefits of the new model 27. Furthermore, the total risk scores of each ICC subject were reckoned in both cohorts, according to the points given for each prognostic factor in the prediction model. Subsequently, a risk classification system was constructed, and the patients were separated into three groups, i.e. the high-, middle-, and low-risk groups. The survival curves were drawn to compare the differences in the survival distribution among the three risk groups.

### Statistical Analysis

The descriptive results of the population studied were expressed as frequencies and percentages across treatment groups. A polytomous logistic regression was implemented to estimate clinicopathological features connected with treatment receipt, and to compare each treatment category individually taking the outcome of no treatment category as a reference. The adjusted odds ratios (aORs) with associated 95% confidence intervals (CIs) were used to describe the results of the logistic regression analyses, and OR greater than 1 suggests a higher rate of receiving treatment. Besides, we employed the Kaplan-Meier method for survival analysis stratified by age, and log-rank tests were utilized to evaluate heterogeneity in survival curves. Further Cox regression analyses were adopted to calculate the stratum-specific hazard ratios (HRs) and the corresponding 95% CIs. Comparisons of clinicopathologic variables between the training and validation cohorts were completed using the chi-square test. Also, the optimal cut-off points of the risk classification system were identified with X-tile program 28.

All analyses were performed using SPSS Statistics software 24.0 (IBM Corporation, Chicago, IL), R software 3.6.2 (https://www.rproject.org/), GraphPad Prism 8.0 (San Diego, CA, USA) and X-tile software 3.6.1 (Yale University, New Haven, CT, USA). Results were considered to be statistically significant if two-sided P < 0.05.

## Results

### Patient Characteristics and Treatment Patterns

In total, 1651 elderly patients with ICC diagnosed from 2004 to 2016 were eligible for analysis. Detailed demographics and characteristics across treatment categories are exhibited in Table 1. Patients aged 65-74 years and ≥75 years accounted for 60.9% and 39.1%, respectively of all the cases. The majority of cases were white (n=1312, 79.5%), female (n=872, 52.8%), and married (n=1455, 88.1%). Moreover, less than half of the patients with known cancer grade had poorly differentiated/undifferentiated tumors. A large proportion of patients presented with middle-sized neoplasms (2.1-10.0 cm, n=1263, 76.5%) and non-metastatic disease. Concerning the therapy mode, the most common treatment for elderly patients was chemotherapy alone (n=483, 29.3%), followed by no tumor-directed treatment (n=441, 26.7%), surgery alone (n=316, 19.1%), radiation treatment with or without other types of therapy (n=289, 17.5%), and surgery plus chemotherapy (n=122, 7.4%). Chemotherapy alone remained the most extensive option for patients aged 65-74 years (n=330, 32.8%), while the largest number of patients aged ≥75 years did not accept any cancer therapy (n=235, 36.4%). Meanwhile, patients aged 65-74 years were more likely to undergo surgery in combination with chemotherapy than those aged ≥75 years (10.0% vs 3.3%), but the proportion of surgery alone was similar in both age groups (19.0% vs 19.3%). In addition, clinicopathological characteristics of patients in the training (n=1184) and validation (n= 467) cohorts are summarized in Table S1. Except for race (P=0.022), the other variables were comparable between the two sets (P > 0.05).

### Polytomous Logistic Regression

The results of the polytomous logistic regression are given in Table 2. Compared to the 65-74 years age group, patients ≥75 years of age were less likely to receive treatment. There were no differences for any of treatment categories on the basis of gender, marital status, or tumor size. Additionally, patients with poorly differentiated or undifferentiated tumors had less possibility to undergo surgery alone (aOR=0.590; 95% CI: 0.399-0.872; P=0.008). Distant metastasis was associated with a lower probability of receiving therapy for all treatment categories except chemotherapy alone. However, patients with regional lymph node metastasis were prone to accept therapy in all treatment categories except surgery alone.

### Survival Outcomes of Different Treatments for Elderly Patients

Compared with the age group of 65‐74 years, patients aged ≥75 years had a significantly shorter median OS (9.0 months vs 12.0 months, P = 0.000). The respective 1-, 3- and 5-year OS rates for patients aged 65-74 years were 48.1%, 20.0%, and 13.7%; and 39.7%, 13.0% and 9.4% for those aged ≥75 years (Figure S1).

The results of survival analysis stratified by age across different treatment categories are displayed in Figure S2 as survival curves and in Table 3 as the corresponding adjusted hazard ratios. Also, Table S2 lists the P values for paired comparison of therapeutic methods. Patients 66-74 years of age who underwent surgery alone had a median survival of 30 months, surgery combined with chemotherapy 26 months, radiotherapy with or without other treatment modalities 17 months, chemotherapy alone 10 months, and no therapy 3 months. Nevertheless, the median OS for patients aged ≥75 years was 21, 25, 14, 9, and 4, respectively. The stratified HRs further uncovered that survival among patients undergoing surgery was significantly better than among those receiving chemotherapy alone, radiotherapy, or no treatment in the two age groups, while there were no significant differences in prognosis between patients treated with surgery alone and those treated with surgery plus chemotherapy (both P > 0.05). Besides, patients who did not receive any tumor-directed therapy had the highest risk of death, which was more prominent among patients 65-74 years of age (HR=4.278; 95% CI: 3.292-5.560; P=0.000) in comparison with those aged ≥75 years (HR=4.154; 95% CI: 3.064-5.631; P=0.000).

### Screening for Prognostic Factors for OS

To further explore the independent predictors of prognosis in elderly patients, univariate and multivariate Cox analyses were performed in the training cohort (n= 1184) as presented in Table 4. The univariate survival analysis demonstrated that all variables except race and marital status were markedly associated with OS. Subsequently, relevant factors with P < 0.05 in the univariate analysis were included in the multivariate model. After multivariate analysis, age, gender, grade, tumor size, T stage, N stage, M stage, and treatment remained significant independent prognostic factors for OS (P < 0.05).

### Construction and Validation of the Nomogram

**Figure 2:**
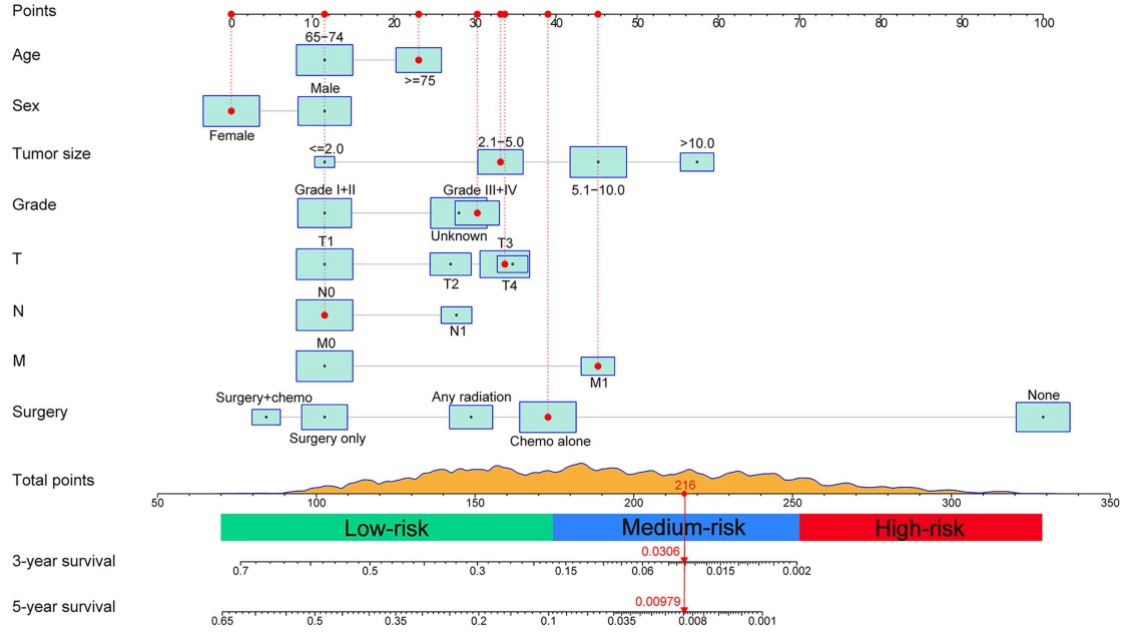
Nomogram for predicting 3- and 5-year overall survival (OS) of patients with intrahepatic cholangiocarcinoma and an example of how to use the nomogram. Each category of the predictors is assigned a score on the Points scale. The sum of these scores is located on the Total points scale and a vertical line is drawn downward to determine the specific probability of 3- and 5-year OS.

A prognostic nomogram integrating all determinants was established for the prediction of OS at 3 and 5 years based on the training set (Figure 2). Each level of the selected variables is assigned a score on the Points scale in the light of its prognostic value, and the total point can be simply acquired by summing the score of each factor. The estimated probability of 3- and 5-year OS corresponding to this total score is determined for each individual elderly patient.

**Figure 3:**
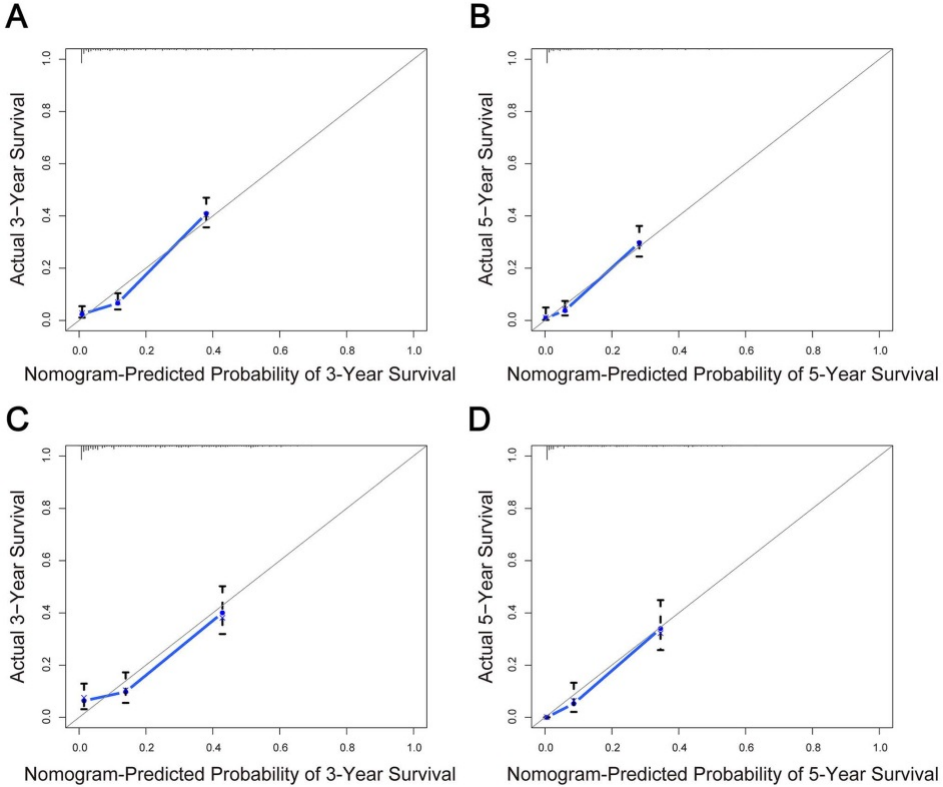
Calibration curves of the nomogram for predicting overall survival (OS) in elderly intrahepatic cholangiocarcinoma patients. Calibration curves for the training cohort at 3 years **(A)** and 5 years **(B)**. Calibration curves for the validation cohort at 3 years **(C)** and 5 years **(D)**. The x-axis represents the nomogram-predicted probability of OS; the y-axis represents the actual OS probability. The plots along the diagonal 45‐degree line indicate a perfect calibration model in which the predicted probabilities are identical to the actual outcomes. Vertical bars indicate 95% confidence intervals.

**Figure 4:**
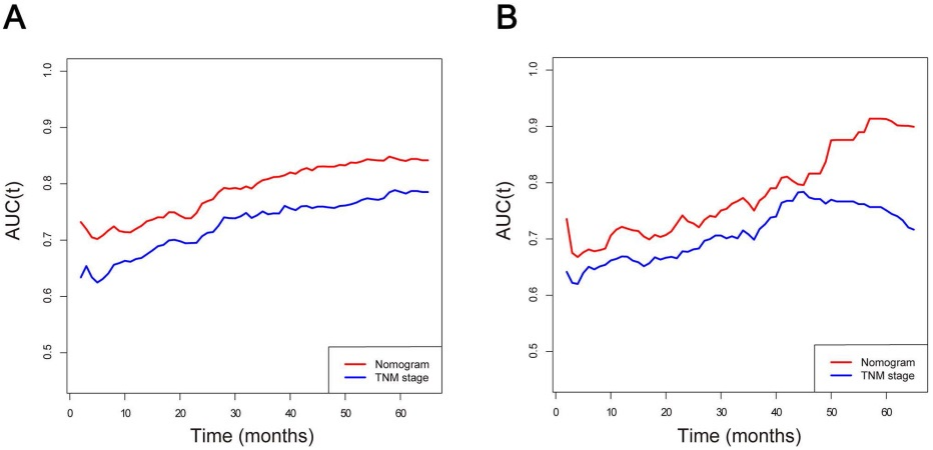
Area under the curve (AUC) models for comparing the predictive ability between the nomogram and TNM stage. **(A)** The nomogram and TNM stage in the training cohort; **(B)** The nomogram and TNM stage in the validation cohort. AUC was calculated for every month from the first to the 60th month.

**Figure 5:**
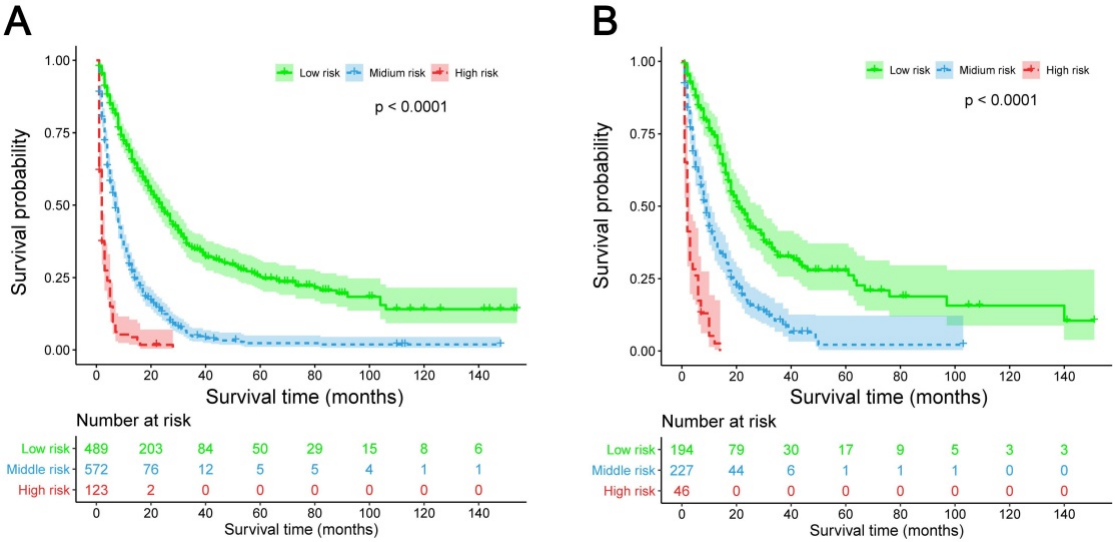
Kaplan-Meier survival curves for three populations (low-risk group, middle-risk group, and high-risk group) of patients classified by prognostic total score calculated from the nomogram in the training cohort **(A)** and validation cohort **(B)**.

The predictive model was then internally validated in the validation dataset. The C-index for prediction of OS was 0.725 (95% CI, 0.705-0.745) in the training set, and 0.724 (95% CI, 0.695-0.753) in the validation set, manifesting accurate capability in prognosis predicting. Meanwhile, the calibration curves for the probability of survival at 3 and 5 years exhibited excellent agreement between the actual and predicted outcomes in both the training cohort and validation cohort (Figure 3). Furthermore, the integrated AUC of the model revealed better discriminative ability in comparison to that of the traditional AJCC TNM staging (training set: 0.842 vs. 0.786, P = 0.009; validation set: 0.913 vs. 0.751, P = 0.000; Figure 4). Importantly, the results of DCA illustrated that our nomogram had larger net benefits and clinical applicability in predicting 3- and 5-year OS than the AJCC stage model (Figure S3). According to the risk classification system, the patients were divided into three groups (low-risk group: 70-174 points; middle-risk group: 175-253 points; high-risk group: 254-328 points). In the training cohort, the median OS time of patients among the three sets were 24, 7, and 2 months, respectively. Similarly, in the validation cohort, the median survival duration for the three risk groups were 21, 9, and 2 months, respectively. And the survival curves between the three risk groups were clearly separated (P < 0.0001, Figure 5), which implied a stronger correlation between the lower risk of patients and the reduction in overall mortality for both cohorts.

### Development of Webserver

Moreover, for the sake of facilitating clinical use, an online version of the nomogram was provided at https://hanlong.shinyapps.io/elderly_icc/ (Figure S4), which can not only predict individualized survival more conveniently and accurately through inputting relevant clinical characteristics, but also avoid errors caused by manual measurement.

## Discussion

With the increased incidence of ICC and the growth of aging population, the management of elderly ICC patients has gradually become a momentous global problem. It is well known that elderly patients tend to perform worse health conditions, malnutrition, reduced performance status, decreased physiological reserve, and more comorbidities, which makes them more vulnerable to stress events and less tolerant to treatments 7. In addition, given the high lethality of ICC, the life expectancy of elderly patients with ICC is more limited even after successful radical operation, resulting in a more negative attitude towards the treatment of elderly ICC patients 8, 9. Based on these premises, the elderly ICC patient population represents a highly heterogeneous group where therapeutic decisions are more complicated. To our knowledge, this study is the first attempt to delineate the treatment paradigms and their prognostic role among a population-based cohort of elderly patients with ICC.

From the results of the current research, less aggressive treatment options such as chemotherapy alone and supportive care were utilized in more than half of all patients (56.0%), and a considerable portion of patients did not accept any tumor-directed therapy even in the early stage of the disease. Additionally, our logistic regression analysis suggested that patients aged ≥75 years preferred to receive supportive care in comparison with patients aged 65-74 years. It seemed conservative for the elderly patient population to choose aggressive treatments, especially among patients aged ≥75 years. Such phenomenon might be explained by the concerns of serious side effects associated with the increase of age in the real world. As indicated earlier, the median survival time and the 3/5 year survival rates of patients aged 75 years and over were worse than those of patients aged 65-74, and older age was recognized as one of the independent poor prognostic factors, which was supported by other studies^18^, ^29^. Consequently, it was recommended to take patient age into consideration when making clinical treatment strategy decisions because of the short expected life.

In addition to age, treatment patterns were also demonstrated to be significantly linked to OS in elderly ICC patients. While some previous studies reported that the risk of complications and mortality after liver surgery increased with age 7, 12, younger and elderly patients undergoing surgical resection for ICC had a similar long-term outcome 7, 14. Likewise, another retrospective study from Canada compared outcomes in older (n=592) and younger (n=321) patients with biliary tract cancers treated with surgery and found no difference in survival between the two groups 30. According to our current research, the prognosis of patients treated with surgery alone or combined treatment was greatly superior to that of patients treated with chemotherapy alone, radiotherapy, or supportive care in the two age groups, which revealed that surgery conferred an obvious survival advantage for elderly patients with ICC. Nevertheless, it was not yet known which subgroups of patients could profit from surgical resection, but a retrospective database cohort study by Tran Cao et al. indicated that surgery, even in the presence of positive lymph nodes, was correlated with a remarkable improvement in OS compared to non-operative therapy 31. This further proofs that surgery, as an active treatment option, should not be abandoned among the selected patients.

The role of adjuvant chemotherapy in resection of ICC remains poorly defined due to the lack of evidence from prospective randomized controlled trials. Some authors held that the application of adjuvant chemotherapy failed to increase survival outcomes and might even have an adverse prognostic impact on elderly ICC patients 7, 9, 32, whereas others believed that the addition of chemotherapy conferred a clear survival benefit, particularly in high-risk patients who had lymph node metastasis or positive surgical margins 33-35. Of note, our study found no significant difference in survival between patients who underwent surgery only and those who underwent surgery plus chemotherapy in both age groups, manifesting that elderly ICC patients did not derive additional survival benefits from adjuvant chemotherapy. The reason for this observation is undoubtedly complex and multi-factorial, but the main one may be that ICC is not very sensitive to chemotherapy drugs. Taken together, adjuvant chemotherapy with increased toxicity may not be suitable for all elderly patients with resectable ICC. However, well-designed prospective clinical studies of high quality in the elderly population are required to corroborate our findings.

Although surgical resection leads to a positive improvement in survival, the majority of cases are unresectable at the time of diagnosis and are not candidates for surgery 36. As expected, chemotherapy alone was the predominant therapy employed in elderly ICC patients. This was consistent with the current National Comprehensive Cancer Network (NCCN) guidelines 37 in which chemotherapy is the preferred form of treatment for metastatic or unresectable disease. Similar to previous reports 3, 9, 36, the use of chemotherapy in both 65-74 years and ≥75 years groups was related to a more favorable survival compared with supportive therapy. As for radiation therapy, its exact efficacy in the treatment of ICC is still uncertain and controversial. Hence, the treatment involving radiotherapy was only utilized in a small proportion of patients. Moreover, our analysis uncovered that in both age groups, radiation therapy helped to prolong survival of patients when compared to no treatment, which was in line with several prior studies 38, 39. Based on these facts, chemotherapy or radiotherapy should not be discarded in a selected group of elderly patients as an aggressive option.

Apart from age and treatment mode, we also determined that patient gender, tumor size, degree of tumor differentiation, and TNM stage were independent predictors of prognosis in elderly ICC patients. All of these easily accessible risk factors were incorporated into a brief nomogram model to better predict outcomes and assist in the development of individualized treatment strategies. Notably, our prognostic nomogram harbored excellent discriminative power and accuracy in both training and validation cohorts based on the findings of C-indexes and calibration curves. More importantly, this nomogram displayed a more accurate prediction for prognosis compared to the conventional AJCC stage system according to AUC analysis (training group: 0.842 vs. 0.786, P = 0.009; validation group: 0.913 vs. 0.751, P = 0.000). Analogously, by adopting DCA, it was fully verified that the established nomogram had higher clinical application value than the AJCC stage. Furthermore, the risk classification system produced by our nomogram could distinguish elderly ICC patients with different levels of risk more accurately and help to provide a more reasonable and appropriate follow-up schedule for patients in different subgroups. Although further well-designed prospective randomized clinical trials are needed to corroborate our observations, we believe that our prognostic model is highly feasible and valuable in estimating personalized clinical outcomes of elderly ICC patients and instructing clinical therapy.

Inevitably, our present study has several limitations that should be considered. First, some important factors affecting the prognosis of the elderly, including health status and comorbidity, were unavailable through the SEER database. The comprehensive geriatric assessment (CGA), as a critical part of treatment decision-making for elderly patients with cancer, was advocated to be used combined with prediction models in the future, which will achieve a better balance between patient survival and living quality^40^, ^41^. Besides, certain additional clinical variables, such as vascular/periductal invasion and serum tumor markers, might also have a potential role in offering prognostic information 4, 7, 32. However, this nomogram did not contain these variables owing to the inherent defects of the database. Third, details about treatment were lacking, such as the order of the therapy, resection margins, the specific chemotherapy and radiation contents, treatment toxicity, and treatment willingness of patients, which should be determined in future research. Fourth, there was a possibility of selection bias in therapeutic strategies because of the nature of a retrospective study design. For instance, the non-random treatment allocation could potentially confound survival analysis. Lastly, we only validated this nomogram internally. Although the predictive model requires continued refinement and improvement, its current form may be useful in assisting clinicians to select optimal treatment decisions and formulate appropriate follow-up strategies. Therefore, it is necessary to utilize follow-up data from other well-defined populations for further evaluation.

## Conclusion

In summary, this study indicated that aggressive treatment should not be discarded, which conferred better prognosis even in patients ≥75 years of age. Moreover, based on independent predictors, the proposed nomogram was pragmatic and reliable for precisely predicting 3-year and 5-year OS, which could help the clinician to provide tailored treatment and to improve prognostic assessment for each patient. Nevertheless, evidence from large-scale prospective validation studies is strongly required to generalize the application of the nomogram.

## Supplementary Material

Supplementary figures and tables.

## Figures and Tables

**Table 1:** The demographics and characteristics of patients according to treatment received.

Characteristic	Total (n=1651) n (%)	Surgery alone (n=316)n (%)	Chemo alone (n=483)n (%)	Radiation alone(n=80)n (%)	None (n=441)n (%)	Surgery+chemo(n=122)n (%)	Surgery+chemo+radiation (n=58) n (%)	Surgery+radiation (n=10)n (%)	Chemo+radiation (n=141)n (%)	


Age, years										
65-74	1005	191 (19.0)	330 (32.8)	38 (3.8)	206 (20.5)	101 (10.0)	49 (4.9)	3 (0.3)	87 (8.7)	
≥75	646	125 (19.3)	153 (23.7)	42 (6.5)	235 (36.4)	21 (3.3)	9 (1.4)	7 (1.1)	54 (8.4)	
Race										
White	1312	247 (18.8)	385 (29.3)	69 (5.3)	332 (25.3)	108 (8.2)	47 (3.6)	8 (0.6)	116 (8.8)	
Non-White	339	69 (20.4)	98 (28.9)	11 (3.2)	109 (32.2)	14 (4.1)	11 (3.2)	2 (0.6)	25 (7.4)	
Gender										
Male	779	153 (19.6)	224 (28.8)	43 (5.5)	193 (24.8)	62 (8.0)	30 (3.9)	4 (0.5)	70 (9.0)	
Female	872	163 (18.7)	259 (29.7)	37 (4.2)	248 (28.4)	60 (6.9)	28 (3.2)	6 (0.7)	71 (8.1)	
Marital status										
Married	1455	276 (19.0)	428 (29.4)	69 (4.7)	394 (27.1)	103 (7.1)	50 (3.4)	8 (0.5)	127 (8.7)	
Unmarried	151	25 (16.6)	46 (30.5)	9 (6.0)	38 (25.2)	15 (9.9)	7 (4.6)	1 (0.7)	10 (6.6)	
Unknown	45	15 (33.3)	9 (20.0)	2 (4.4)	9 (20.0)	4 (8.9)	1 (2.2)	1 (2.2)	4 (8.9)	
Grade										
Well/Moderately differentiated	580	188 (32.4)	125 (21.6)	21 (3.6)	111 (19.1)	66 (11.4)	32 (5.5)	7 (1.2)	30 (5.2)	
Poorly differentiated/Undifferentiated	409	93 (22.7)	118 (28.9)	8 (2.0)	96 (23.5)	45 (11.0)	21 (5.1)	2 (0.5)	26 (6.4)	
Unknown	662	35 (5.3)	240 (36.3)	51 (7.7)	234 (35.3)	11 (1.7)	5 (0.8)	1 (0.2)	85 (12.8)	
Tumor size, cm										
≤2.0	102	26 (25.5)	19 (18.6)	1 (1.0)	26 (25.5)	10 (9.8)	12 (11.8)	1 (1.0)	7 (6.9)	
2.1-5.0	499	120 (24.0)	107 (21.4)	28 (5.6)	141 (28.3)	43 (8.6)	19 (3.8)	2 (0.4)	39 (7.8)	
5.1-10.0	764	135 (17.7)	245 (32.1)	40 (5.2)	193 (25.3)	51 (6.7)	22 (2.9)	7 (0.9)	71 (9.3)	
>10.0	286	35 (12.2)	112 (39.2)	11 (3.8)	81 (28.3)	18 (6.3)	5 (1.7)	0 (0.0)	24 (8.4)	
T stage										
T1	655	146 (22.3)	163 (24.9)	45 (6.9)	194 (29.6)	40 (6.1)	14 (2.1)	3 (0.5)	50 (7.6)	
T2	332	87 (26.2)	86 (25.9)	4 (1.2)	77 (23.2)	37 (11.1)	16 (4.8)	3 (0.9)	22 (6.6)	
T3	483	45 (9.3)	180 (37.3)	27 (5.6)	127 (26.3)	34 (7.0)	19 (3.9)	3 (0.6)	48 (9.9)	
T4	181	38 (21.0)	54 (29.8)	4 (2.2)	43 (23.8)	11 (6.1)	9 (5.0)	1 (0.6)	21 (11.6)	
N stage										
N0	1267	279 (22.0)	342 (27.0)	65 (5.1)	351 (27.7)	81 (6.4)	41 (3.2)	9 (0.7)	99 (7.8)	
N1	384	37 (9.6)	141 (36.7)	15 (3.9)	90 (23.4)	41 (10.7)	17 (4.4)	1 (0.3)	42 (10.9)	
M stage										
M0	1222	301 (24.6)	279 (22.8)	60 (4.9)	300 (24.5)	114 (9.3)	57 (4.7)	9 (0.7)	102 (8.3)	
M1	429	15 (3.5)	204 (47.6)	20 (4.7)	141 (32.9)	8 (1.9)	1 (0.2)	1 (0.2)	39 (9.1)	

**Table 2:** Polytomous logistic regression for each treatment group (vs. no therapy) as the dependent variable of interest.

Characteristic	Surgery alone vs. notreatment, OR (CI)	P value	Chemo alone vs. notreatment, OR (CI)	P value	Any radiation vs. no treatment,OR (CI)	P value	Surgery+chemo vs. no treatment, OR (CI)	P value	


Age, years									
65-74	Ref		Ref		Ref		Ref		
≥75	0.618 (0.446-0.856)	0.004	0.422 (0.320-0.556)	0.000	0.570 (0.418-0.778)	0.000	0.204 (0.120-0.346)	0.000	
Race									
White	Ref		Ref		Ref		Ref		
Non-White	0.881 (0.600-1.292)	0.515	0.751 (0.546-1.033)	0.079	0.615 (0.420-0.901)	0.013	0.380 (0.202-0.713)	0.003	
Gender									
Male	Ref		Ref		Ref		Ref		
Female	0.785 (0.568-1.085)	0.143	1.003 (0.766-1.313)	0.985	0.773 (0.569-1.051)	0.100	0.811 (0.523-1.257)	0.348	
Marital status									
Married	Ref		Ref		Ref		Ref		
Unmarried	0.869 (0.490-1.542)	0.632	1.083 (0.679-1.727)	0.737	1.035 (0.609-1.759)	0.899	1.461 (0.731-2.920)	0.283	
Unknown	2.783 (1.058-7.319)	0.038	1.095 (0.421-2.848)	0.853	1.787 (0.662-4.827)	0.252	2.133 (0.566-8.033)	0.263	
Grade									
Well/Moderately differentiated	Ref		Ref		Ref		Ref		
Poorly differentiated/Undifferentiated	0.590 (0.399-0.872)	0.008	0.976 (0.665-1.432)	0.902	0.679 (0.437-1.055)	0.085	0.761 (0.462-1.254)	0.285	
Unknown	0.100 (0.064-0.154)	0.000	0.902 (0.653-1.245)	0.529	0.783 (0.549-1.116)	0.176	0.090 (0.045-0.180)	0.000	
Tumor size, cm									
≤2.0	Ref		Ref		Ref		Ref		
2.1-5.0	0.834 (0.430-1.617)	0.591	1.100 (0.571-2.122)	0.776	0.816 (0.427-1.560)	0.538	0.738 (0.306-1.780)	0.498	
5.1-10.0	0.952 (0.491-1.846)	0.885	1.751 (0.919-3.335)	0.088	0.971 (0.511-1.844)	0.928	0.828 (0.342-2.003)	0.675	
>10.0	0.569 (0.268-1.210)	0.143	1.661 (0.839-3.290)	0.146	0.558 (0.272-1.142)	0.110	0.553 (0.206-1.480)	0.238	
T stage									
T1	Ref		Ref		Ref		Ref		
T2	1.622 (1.066-2.469)	0.024	1.263 (0.857-1.859)	0.238	0.993 (0.633-1.556)	0.975	2.011 (1.137-3.555)	0.016	
T3	0.616 (0.392-0.968)	0.036	1.253 (0.898-1.750)	0.185	1.401 (0.955-2.055)	0.084	1.253 (0.702-2.237)	0.445	
T4	1.778 (1.026-3.083)	0.040	1.211 (0.755-1.943)	0.427	1.593 (0.944-2.686)	0.081	1.253 (0.557-2.819)	0.586	
N stage									
N0	Ref		Ref		Ref		Ref		
N1	0.620 (0.393-0.978)	0.040	1.413 (1.027-1.943)	0.033	1.455 (1.008-2.100)	0.045	2.107 (1.288-3.449)	0.003	
M stage									
M0	Ref		Ref		Ref		Ref		
M1	0.130 (0.073-0.232)	0.000	1.369 (1.029-1.822)	0.031	0.500 (0.348-0.718)	0.000	0.152 (0.070-0.329)	0.000	

OR >1 indicates higher odds of receiving treatment. CI, confidence interval; OR, odds ratio; Ref, reference.

**Table 3:** Adjusted hazard ratio for different treatment in elderly patients according to age groups.

Treatment	65-74 years			≥75 years		
	HR	95% CI	P value	HR	95% CI	P value
Surgery alone	Ref			Ref		
Surgery + Chemo	0.839	0.600-1.175	0.307	1.017	0.561-1.843	0.957
Chemo only	1.647	1.271-2.134	0.000	1.960	1.422-2.701	0.000
Any radiation	1.324	1.007-1.741	0.045	1.565	1.116-2.194	0.009
No therapy	4.278	3.292-5.560	0.000	4.154	3.064-5.631	0.000

95% CI, 95% confidence interval; HR, hazard ratio; Ref, reference.

**Table 4:** Univariate and multivariate Cox regression analyses of overall survival in the training set.

Variable	Univariate analysis			Multivariate analysis		
	HR	95% CI	P value	HR	95% CI	P value
Age, years			0.001			0.011
65-74	Ref			Ref		
≥75	1.256	1.100-1.435	0.001	1.197	1.042-1.375	0.011
Race			0.370			
White	Ref					
Non-White	1.075	0.918-1.258	0.370			
Gender			0.046			0.010
Male	Ref			Ref		
Female	0.875	0.768-0.997	0.046	0.840	0.736-0.959	0.010
Marital status			0.185			
Married	Ref					
Unmarried	0.888	0.703-1.120	0.315			
Unknown	0.710	0.465-1.084	0.113			
Grade			0.000			0.001
Well/Moderately differentiated	Ref			Ref		
Poorly differentiated/Undifferentiated	1.534	1.288-1.826	0.000	1.342	1.124-1.601	0.001
Unknown	1.750	1.501-2.040	0.000	1.288	1.096-1.513	0.002
Tumor size, cm			0.000			0.000
≤2.0	Ref			Ref		
2.1-5.0	1.344	0.955-1.890	0.090	1.407	0.998-1.982	0.051
5.1-10.0	1.686	1.211-2.349	0.002	1.676	1.195-2.350	0.003
>10.0	2.261	1.587-3.220	0.000	2.026	1.408-2.916	0.000
T stage			0.000			0.000
T1	Ref			Ref		
T2	1.082	0.894-1.309	0.416	1.272	1.047-1.545	0.015
T3	1.668	1.424-1.953	0.000	1.416	1.191-1.682	0.00
T4	1.710	1.380-2.119	0.000	1.456	1.167-1.816	0.001
N stage			0.000			0.002
N0	Ref			Ref		
N1	1.469	1.257-1.716	0.000	1.288	1.094-1.517	0.002
M stage			0.000			0.000
M0	Ref			Ref		
M1	2.243	1.938-2.595	0.000	1.669	1.423-1.958	0.000
Treatment			0.000			0.000
No therapy	Ref			Ref		
Surgery alone	0.212	0.171-0.262	0.000	0.251	0.200-0.315	0.000
Chemo only	0.506	0.428-0.599	0.000	0.399	0.334-0.476	0.000
Any radiation	0.356	0.291-0.435	0.000	0.340	0.276-0.419	0.000
Surgery + Chemo	0.204	0.150-0.278	0.000	0.226	0.164-0.311	0.000

95% CI, 95% confidence interval; HR, hazard ratio; Ref, reference.

## Abbreviations

CCA: Cholangiocarcinoma
ICC: Intrahepatic cholangiocarcinoma
AJCC: American Joint Committee on Cancer
TNM: tumor-node-metastasis
SEER: Surveillance, Epidemiology, and End Results
NCI: National Cancer Institute
ICDO-3: International Classification of Diseases for Oncology, 3rd Edition
OS: Overall survival
C-index: concordance index
ROC: receiver operating characteristic
AUC: area under receiver operating characteristic curve
DCA: decision curve analysis
aORs: adjusted odds ratios
CIs: confidence intervals
HRs: hazard ratios
NCCN: National Comprehensive Cancer Network
CGA: comprehensive geriatric assessment
