# Supplementary material for: Describing Treatment Patterns for Elderly Patients with Intrahepatic Cholangiocarcinoma and Predicting Prognosis by a Validated Model: A Population-Based Study
Source: J Cancer. 2021 Mar 30;12(11):3114–25. doi: 10.7150/jca.53978 (PMC8100797; doi:10.7150/jca.53978)
Supplement: Supplementary file 1 — Supplementary figures and tables. [file jcav12p3114s1.pdf]

Supplementary information for

Describing Treatment Patterns For Elderly Patients With Intrahepatic Cholangiocarcinoma And Predicting Prognosis By a Validated Model: A Population-Based Study

Supporting Tables

Table S1 The demographic and clinicopathological variables of the training set and validation set.

Table S2 P values for paired comparison of therapeutic methods according to age.

Supporting Figures

Figure S1 The 1-, 3- and 5-year overall survival rates for elderly patients in 65-74 years and ≥75 years groups.

Figure S2 Overall survival of elderly patients diagnosed with intrahepatic cholangiocarcinoma according to type of treatment. (A) Representing patients aged 65-74 years; (B) Representing patients aged ≥75 years. All curves represent actual survival evaluated by Kaplan-Meier.

Figure S3 Decision curve analysis (DCA) for the Nomogram and TNM stage in prediction of prognosis at 3-year (A) and 5-year (B) point in the training cohort. DCA for the Nomogram and TNM stage in prediction of prognosis at 3-year (C) and 5-year (D) point in the validation cohort.

Figure S4 Layout of an online version of the developed nomogram (https://hanlong.shinyapps.io/elderly_icc/).

Table S1 The demographic and clinicopathological variables of the training set and validation set.

Variable	Training set (n=1184)	Validation set (n=467)	P value
Age, years			0.676
65-74	717 (60.6)	288 (61.7)	
≥75	467 (39.4)	179 (38.3)	
Race			0.022
White	924 (78.0)	388 (83.1)	
Non-White	260 (22.0)	79 (16.9)	
Gender			0.856
Male	557 (47.0)	222 (47.5)	
Female	627 (53.0)	245 (52.5)	
Marital status			0.834
Married	1041 (87.9)	414 (88.7)	
Unmarried	109 (9.2)	42 (9.0)	
Unknown	34 (2.9)	11 (2.4)	
Grade			0.584
Well/Moderately differentiated	425 (35.9)	155 (33.2)	
Poorly differentiated/Undifferentiated	290 (24.5)	119 (25.5)	
Unknown	469 (39.6)	193 (41.3)	
Tumor size, cm			0.508
≤2.0	71 (6.0)	31 (6.6)	
2.1-5.0	359 (30.3)	140 (30.0)	
5.1-10.0	558 (47.1)	206 (44.1)	
>10.0	196 (16.6)	90 (19.3)	
T stage			0.405
T1	455 (38.4)	200 (42.8)	
T2	242 (20.4)	90 (19.3)	
T3	352 (29.7)	131 (28.1)	
T4	135 (11.4)	46 (9.9)	
N stage			0.486
N0	914 (77.2)	353 (75.6)	
N1	270 (22.8)	114 (24.4)	
M stage			0.966
M0	876 (74.0)	346 (74.1)	
M1	308 (26.0)	121 (25.9)	
Treatment			0.991
Surgery alone	229 (19.3)	87 (18.6)	
Chemo only	347 (29.3)	136 (29.1)	
Any radiation	204 (17.2)	85 (18.2)	
No therapy	316 (26.7)	125 (26.8)	
Surgery + Chemo	88 (7.4)	34 (7.3)	

Table S2 P values for paired comparison of therapeutic methods according to age.

	Treatment	Surgery alone	Surgery + Chemo	Chemo only	Any radiation	No therapy
65-74 years	Surgery alone	——	0.977	0.000	0.000	0.000
	Surgery + Chemo	0.977	——	0.000	0.001	0.000
	Chemo only	0.000	0.000	——	0.000	0.000
	Any radiation	0.000	0.001	0.000	——	0.000
	No therapy	0.000	0.000	0.000	0.000	——
≥75 years	Surgery alone	——	0.714	0.000	0.000	0.000
	Surgery + Chemo	0.714	——	0.001	0.067	0.000
	Chemo only	0.000	0.001	——	0.004	0.000
	Any radiation	0.000	0.067	0.004	——	0.000
	No therapy	0.000	0.000	0.000	0.000	——

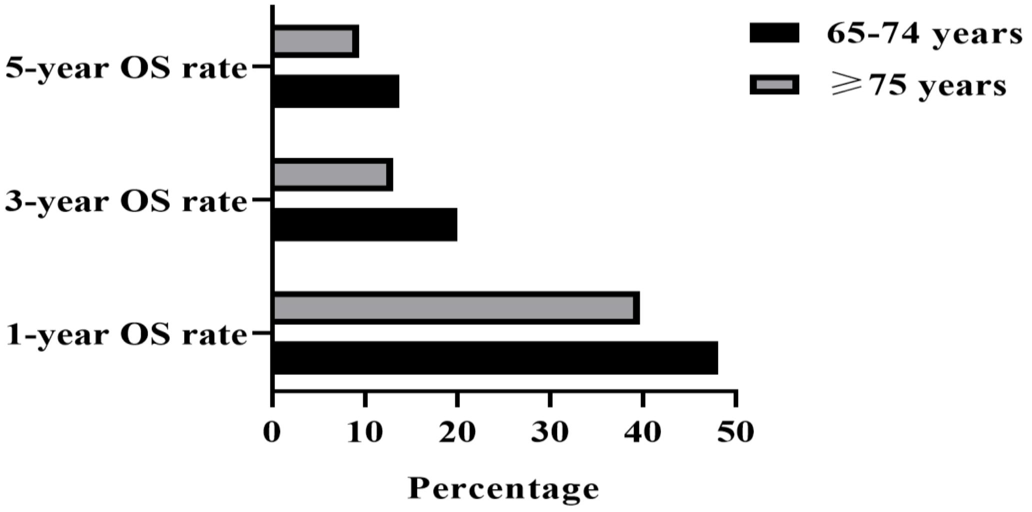

Figure S1 The 1-, 3- and 5-year overall survival rates for elderly patients in 65-74 years and ≥75 years groups.

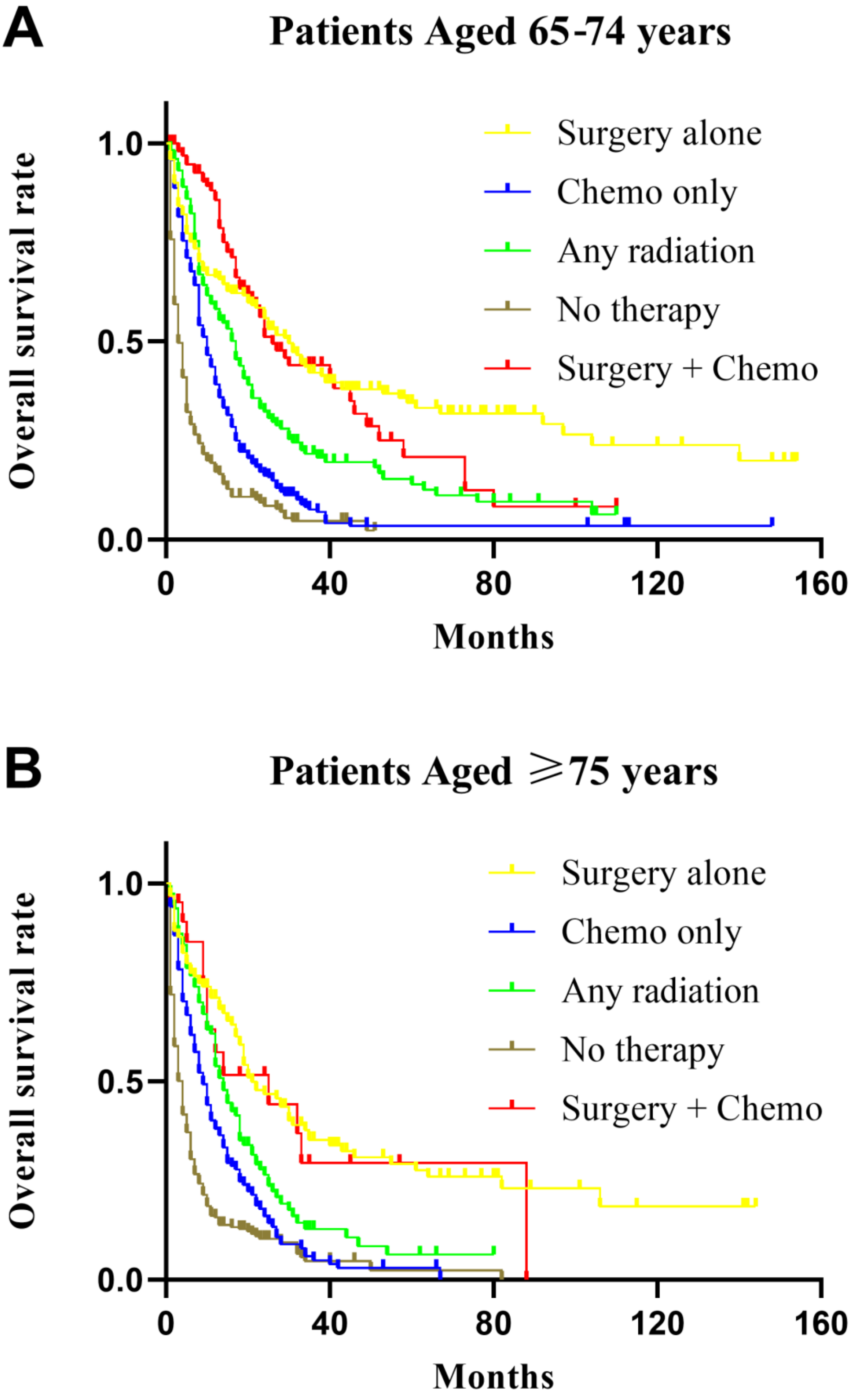

Figure S2 Overall survival of elderly patients diagnosed with intrahepatic cholangiocarcinoma according to type of treatment. (A) Representing patients aged 65-74 years; (B) Representing

patients aged ≥75 years. All curves represent actual survival evaluated by Kaplan-Meier.

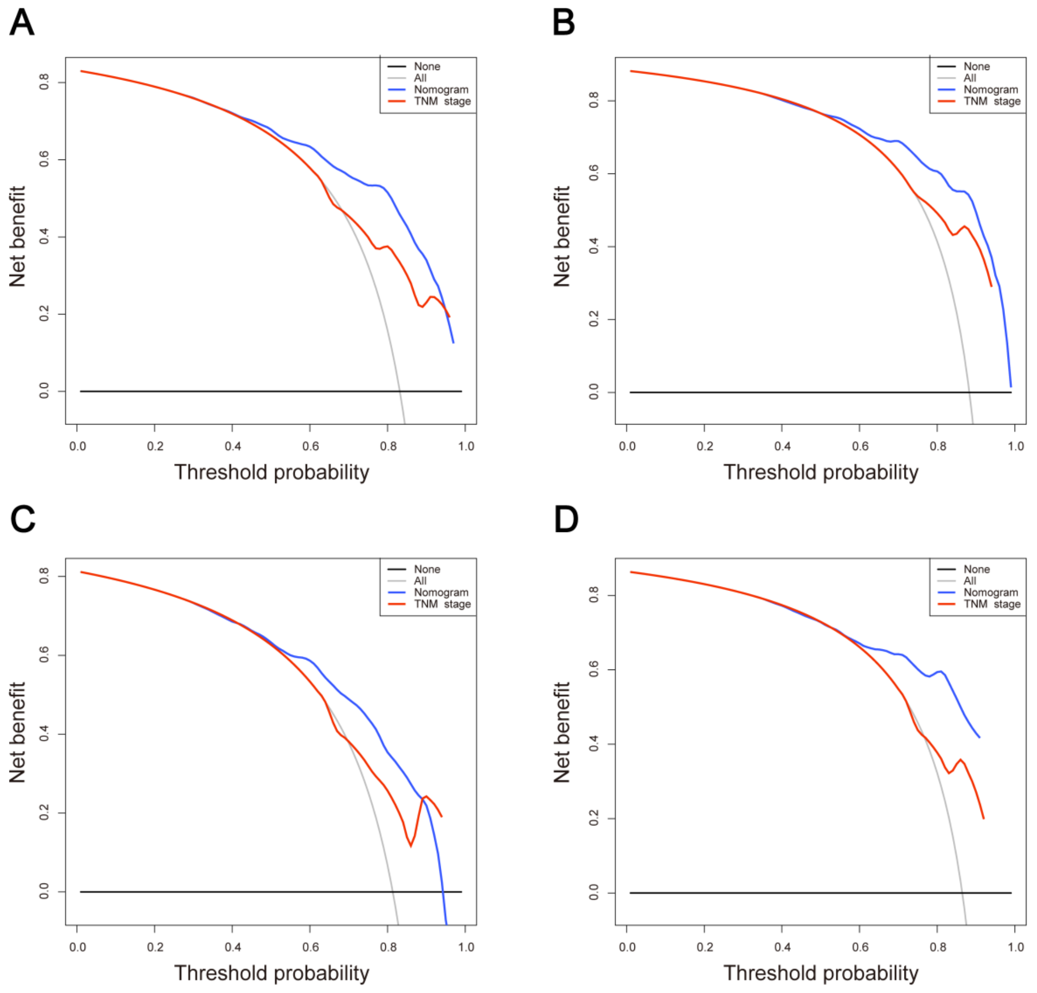

Figure S3 Decision curve analysis (DCA) for the Nomogram and TNM stage in prediction of prognosis at 3-year (A) and 5-year (B) point in the training cohort. DCA for the Nomogram and TNM stage in prediction of prognosis at 3-year (C) and 5-year (D) point in the validation cohort.

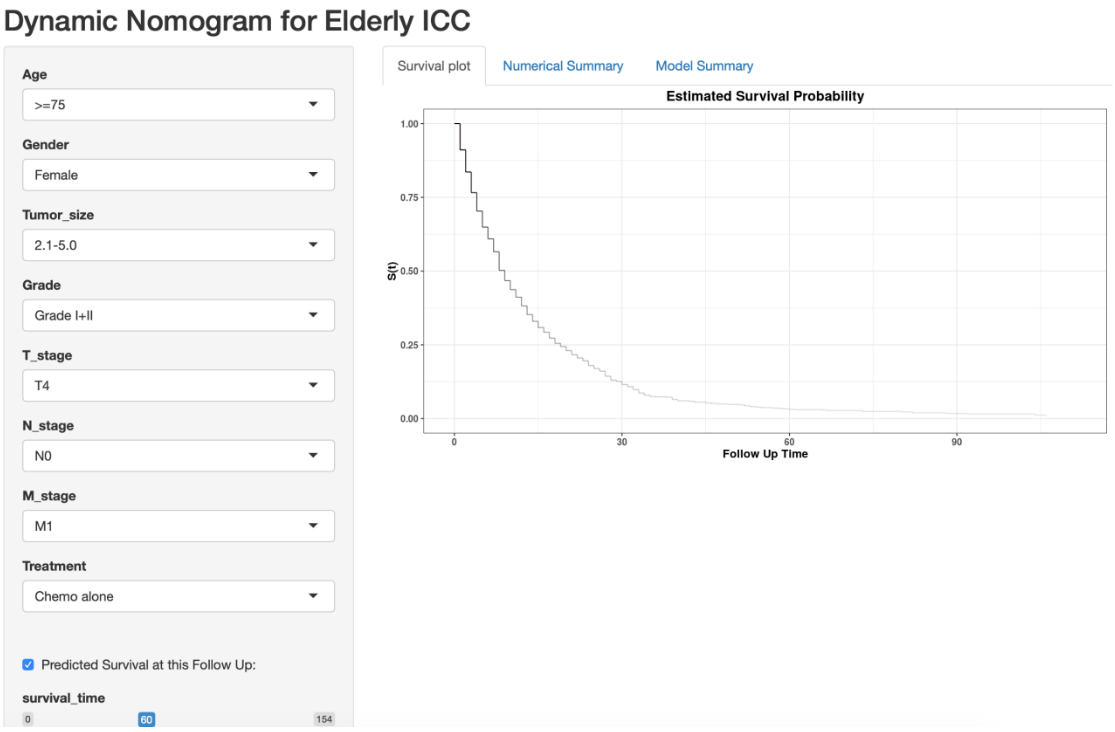

Figure S4 Layout of an online version of the developed nomogram (https://hanlong.shinyapps.io/elderly_icc/).
